# On the Nature and Pathogeny of Malignant Jaundice According to Recent Works

**Published:** 1880-10

**Authors:** Albert Mathieu

**Affiliations:** Interne des hôpitaux (Paris)


### Original Translations.

### ON THE NATURE AND PATHOGENY OF MALIGNANT JAUNDICE, ACCORDING TO RECENT WORKS. By ALBERT MATHIEU, interne des hôpitaux (Paris).

(Translated for the CHICAGO MEDICAL JOURNAL AND EXAMINER by H. D. VALIN, M. D., Chicago.

Dr. Rendu, in an excellent article in the *Dictionnaire de Dechambre*, appropriately gave the study of malignant jaundice the first place in the pathology of the liver. In fact, malignant jaundice is no longer considered a well-defined disease, existing *per se*, but as a set of symptoms met in several affections.

"We do not look upon malignant jaundice as a special disease, but simply as a complication which may take place during the course of any disease of the liver, a common ultimate phase of any degeneration of that gland, which may yet result in a cure." (Rendu.)

This is very different from the signification formerly given to the term malignant jaundice, or to its synonyms, hæmorrhagic, typhoid, fatal icterus, acute yellow atrophy of the liver.

The essential disease formerly described under that name does exist, but its only characteristics are a rapid course, a toxic cause, probably, and anatomically a quick destruction of the active elements of the liver. These symptoms and pathological lesions can all be found in a variety of pathological conditions, with a more or less chronic course. The nervous phenomena, hæmorrhages and prostration, are symptoms common to many affections. Shall we say, however, that the complexus of symptoms are indifferently produced in all general and local diseases affecting the liver, without a corresponding and pathogenic cause being found in that viscus? On the contrary, works lately published aim at demonstrating that the sum of the phenomena referred to have always their origin in the same lesion—the destruction of the liver cells, whatever the cause of such a destruction may be. In the same manner that a relaxation of the vascular system results from various affections of the heart, malignant jaundice is the last result of destructive diseases of the liver. Let us now add another important character. It seems that the suppression of liver cells is not the first step, although that was the opinion held at first, as it is even yet by a few. It is more likely the result either of a deep change in the organism, manifested by a pathological change in the blood, or a formative process ending as it were in the mechanical suppression of the active elements of the liver.

This view concerning malignant icterus clearly results from a perusal of late works on the subject. The article of Dr. Rendu in the Cyclopedic Dictionary, the chapter on acute yellow atrophy of Thierfelder in Ziemssen's Handbook, and the inaugural thesis of Dr. Mossé, have been our chief guide. Their interesting perusal incites one to think that science actually tends to strengthen that pathological point, and although some obscure corners remain, one can form a satisfactory general idea of the phenomena designed by the terms malignant jaundice or yellow atrophy.

We must presently explain these improper terms. Jaundice is not a necessary concomitant of the suppression of liver cells, and the chief features of the complexus of symptoms, hæmorrhages and cerebral manifestations, have nothing to do directly with the absorption of bile in the blood. The importance of the morbid phenomena is not at all in ratio to the intensity of the jaundice. Some medical writers, struck by the marked diminution in the size of the liver, and taking such a degeneration as pathognomonic, called acute yellow atrophy, this affection named by others malignant, fatal, essentially hæmorrhagic icterus, etc. But malignant jaundice may be present without any atrophy of the liver, or when the latter is atrophied, if the diminution in size is produced very slowly. It is necessary, however, being agreed on that point, to continue to give to that sum of symptoms now the name of malignant jaundice, and now that of yellow atrophy. We prefer the former term, although the icterus may be absent sometimes, rather than to conjecture as to the true nature of things.

It may be proper, to facilitate the understanding of this review, to give here the plan which we propose to follow, or, rather, the consecutive propositions which might form a conclusion to this article.

1. The absorption of the constituents of bile in the blood is not apt to give rise to malignant jaundice, and, accordingly, theories based on cholemia, acholia, etc., are groundless.

2. Neither is malignant jaundice due to uræmia, and, should the kidney have something to do with the pathology of that complexus of symptoms, it is not of a primary importance.

3. The destruction of liver cells is the only anatomical characteristic. This destruction may be produced under the influence of widely different causes, which may be divided in two chief classes—mechanical and toxic. The former produce a slow destruction of the active elements of the liver, the latter a quick destruction.

4. There exist, however, as demonstrated by phosphorus poisoning, transitory forms between slow and mechanical destruction, and the rapid destruction depending on a toxic cause.

5. The peculiar state of the blood and the existence of epidemics show that a general poisoning precedes the hepatic atrophy, in genuine cases, in the same manner that the absorption of phosphorus precedes the signs of poisoning by that substance.

6. Malignant jaundice, contrarily to what has long been the common belief, may terminate in a cure.

The physicians who first described malignant jaundice have been impressed by two phenomena chiefly—the presence of jaundice and the constant occurrence of lesions in the liver. Very naturally, they gave the jaundice the greatest importance. Its appearance, indeed, most frequently precedes the occurrence of nervous manifestations and hæmorrhages. Hence, the hepatic lesions must have been influenced by the jaundice, the bile retained in the secreting canals, or, secreted in excess, dissolving the liver cells. (Henoch, von Dusch, Rokitansky.) This, however, was proven to be false. (Robin, Kühne, Wickham Legg.) The bile, no more than the biliary acids, has not the natural property to destroy the liver cells.

It was supposed that the constiuents of bile introduced or retained in the circulation would produce on the blood a particularly poisonous effect. Hence, the symptoms were attributed to cholemia (Piorry), choletoxemia (Lebert), acholia (Frerichs). It has been demonstrated that, although the presence of bile in the blood is not harmless, it is not capable of producing malignant jaundice.

Researches in experimental pathology demonstrate that it is impossible to give rise to malignant jaundice by artificially introducing bile into the blood, even in a large quantity, provided, however, that the bile be well filtered. Finally, very significant pathological facts prove that there is no necessary connection between the jaundice and the nervous accidents.

Malignant jaundice may supervene towards the end of common cirrhosis, even if the latter has progressed unaccompanied with jaundice (Thierfelder). It is a question, however, why those nervous phenomena are not developed more frequently in cases of intense and prolonged icterus, why, in a word, all cases of jaundice do not have to a certain extent the typhoidal aspect, if cholemia sufficed to explain cerebral disorders.

The theory of acholia cannot be sustained, because the biliary acids do not pre-exist in the blood. Not only are they excreted, but they are also secreted by the liver.

Moleschott, Lehman and Kunde, after removing the liver in frogs, and keeping the animals alive during several weeks, have not found biliary salts in their blood.

Is there a resorption of bile after secretion and bile-poisoning?

Such a poisoning might be dependent on the coloring matter, the biliary acids, or cholesterine.

For what relates to the coloring matter, it is more and more generally admitted that bilirubine, the prototype of the various coloring matters of bile, is derived from hæmoglobine, that beside a little modification the blood and the bile, pigments are the same. The chemical demonstration of that point is imperfect as yet, although very clear in a clinical point of view, and it is from that circumstance and from that identity in nature that the theory that icterus arises from hæmaphæin (the brown coloring matter of the blood) was founded, and it has been sustained in France by Gubler, and ably exposed by Dreyfus Brisach in his inaugural thesis. *Hœmaphœic* icterus may often accompany grave affections as a result but not as a cause.

Are the symptoms of malignant jaundice depending on biliary salts as some pretend? They have been injected in a large quantity into the blood without giving rise to characteristic nervous phenomena. This was the result of the experiments of Traube, Müller, Felz and Ritter, Vulpian, etc. Whenever the injection is considerable, the results hardly differ from those obtained by injecting water; or at times symptoms pertaining more to simple jaundice are produced: slowness of the pulse, lowering of the temperature, emesis, diarrhœa. At times hæmaturia appeared, and sometimes ptyalism.

In any case, biliary salts must be reckoned harmless. Their presence in the blood cannot determine the occurrence of malignant jaundice.

Icterus may long exist without maglignant jaundice, as extensively proven by Dr. Strauss in his admission thesis. Whenever malignant jaundice supervenes in the course of chronic jaundice, it is indirectly, and through the inflammation of the biliary canaliculi, and through the interstitial hepatitis resulting therefrom, as is the case in hypertrophic cirrhosis. These accidents must again be referred to the inflammatory and destructive process rather than to a simple resorption of bile.

It remains to examine the hypothesis of cholesteræmia proposed by Dr. Austin Flint.

According to that author, the symptoms of malignant jaundice would be owing to the retention and the accumulation of cholesterine in the blood. In a normal state, in an adult, the blood contains 0.445 gr. to 0.750 gr. of cholesterine. In a case of cirrhosis of the liver, with nervous manifestations, he found 0.922 gr., in another case 1.850 gr. Pagés also found 1.85 gr., and Picot 1.864 gr.

Cholesterine has been injected into the blood and has never reproduced the grave manifestations of malignant jaundice.

Pagés and Felz obtained only negative results. R. Müller has determined a weakening of the pulse and even coma: the cholesterine which he used being porphyrised and undissolved so that cerebral emboli were probably produced.

It seems now well demonstrated that malignant jaundice is not caused by the absorption of the biliary constituents in the blood. This is obvious from physiological experiments and still more from the true existence of the symptoms of malignant jaundice without icterus. Not only is its absence met in chronic cirrhosis, and slowly destructive affections ; but Bamberger has recorded a case of acute yellow atrophy without jaundice (Krankh. des Chylopoë. Syst. 2 Aufl. p. 532). It referred to the case of a woman thirty years old who was seized with delirium and mania on the day following her confinement. The autopsy revealed an acute yellow atrophy of the liver far advanced; hardly a few cells had remained intact. Similar facts have been recorded by Eppinger and Liebermeister.

What neither bile nor its constituents can do may be produced by urea, according to others. We thus approach the renal theory of malignant jaundice lately sustained by Dr. Decandin.

Because renal lesions are frequent, even in simple jaundice, it does not follow that the nervous manifestations should be attributed to uræmia. It is doubtless very important to have healthy kidneys, capable of eliminating the bile absorbed in the blood; it is doubtless very useful to have a sieve to filter the leucine, the tyrosine and the other noxious substances apt to occasion poisoning, but it is going too far to consider the kidney as the starting point of the symptoms of poisoning, and above all to identify malignant icterus with uræmia.

Dr. Decandin resumed towards the last of his thesis, the objections to the uræmic theory; it was helping the critic, still he does not seem to have answered these objections.

Albuminuria does not accompany malignant jaundice; nor is the temperature lowered. There is no œdema nor puffiness of the face, no albuminuric retinitis, nor hypertrophy of the heart.

Moreover, the convulsive fits of uræmia do not resemble the crises of malignant icterus in which hardly any eclampsia is noticed.

In malignant jaundice, delirium, nervousness, later stupor and coma are prominent. Says Traube: the fits have something "psychiatric."

Two very curious observations might form a basis to the uræmic theory. One is from Dr. Vallin, the other from Dr. Bouchard.

In that recorded by Dr. Vallin, there were all the signs of malignant jaundice though the autopsy revealed no lesion of the liver, while the kidney on the other hand, presented a marked degree of fatty degeneration. This at first sight seems very demonstrative. Nevertheless, hepatic lesions, appreciable to the eye or the microscope, have been wanting in several other observations. It does not prove that the liver cells were intact, for Dr. Quinquand has recently demonstrated in a similar case that the tissue of the liver, healthy in appearance, showed a large quantity of leucine and tyrosine when chemically analyzed. It is very interesting to join the result to what was already known of the appearance of those extractive matters in the liver as well as in the blood and the urine.

It seems in reality, and ulterior experiments are likely to confirm such results, that the chemical lesion is the leading one, and that the hepatic cells have been physiologically suppressed, although their morphological texture did not seem altered in the least.

The observation of Dr. Bouchard is very instructive. It shows the possibility of curing malignant jaundice. In the first stage the elimination of urea was strikingly increased. In a second stage the urine and the urea had been notably diminished and the nervous manifestations of malignant icterus appeared. At last the cure was ushered in by a marked increase in the quantity of urine and urea, a critical phenomenon which has also been observed since by Mossé, Arnould, and Coyne. This fact, however, can hardly be brought to uphold the theory of uræmia. Indeed, cases are not rare which, at first presenting an augmentation of urea, later showed a marked diminution; but there was probably a greater diminution in the production of urea than in its excretion. And this agrees well with what Meissner, Bronardell, and Murchisson have taught concerning the office of the liver in the production of urea. During the period of active congestion, there is an excess of urea produced; during that of atrophy, it disappears.

Arnould and Coyne lately analyzed the blood in malignant jaundice and found the quantity of urea diminished. Then it may be admitted that although uræmia may accompany malignant icterus, it is not identical with it, and Rendu and Vulpian rightly claim for the kidney an important, yet accessory and secondary office, in the pathology of malignant jaundice. Dr. Decandin brings to the help of his theory the researches, full of interest, of Julius Möbins. This observer found, chiefly after prolonged jaundice, some biliary coloring matter accumulated in the tubuli, and degenerated epithelium in the convoluted tubes. But nothing indicates that these lesions ever determined nervous phenomena similar to those of malignant icterus, although this would be the important point to be proven.

A very satisfactory observation of Dr. Rendu can be found at the end of Dr. Mossé's thesis, under the head of aggravated jaundice. A man sixty years old was suddenly attacked with prostration, and three days later with jaundice. A comparatively large quantity of albumen was found in the urine. This latter was greenish, leaving a muco-purulent deposit. Patient fell in a low stage, dying with asthenia. Liver was normal; there was a pyelo-nephritis with increase in the size of the kidneys. There is a fine example of the result of icterus in a man whose kidneys were out of order. But as Dr. Rendu remarked, this was not a genuine case of malignant jaundice. The same could be said of other forms of aggravated icterus.

This leads us to place in the first rank the lesions of the liver, whose nature and pathogeny are now to be explained.

We borrow from Dr. Mossé an effort at a classification of the lesions of the liver. Little need be added to it to show that there is an insensible gradation from the lesions of typical, acute yellow atrophy, and those of chronic atrophy, of the cirrhosis of Laennec, passing through acute diffuse interstitial hepatitis.

"*First Period.*—Congestion of liver, increase in size, a lobular aspect appears on cutting. Tumefaction, anomaly. (The lesions observed in all malignant fevers). Disease may stop here, or pass unobserved, patient recovering.

"*Second Period.*—The consistency and the size of the liver begins to diminish in a general way, and in equal proportions. The hue, on cutting, is more or less yellowish and marbled. The normal granular aspect is lost.

"Anæmia of the parenchyma, hæmorrhage at times. The microscope reveals tumefaction and anomaly. Appearance of fatty globules and sometimes of biliary pigment in the interior of cells; some begin to take on atrophy, others may even be entirely disintegrated; protein granulations. The interlobular space is increased (albumino-fibrinous exudation, Frerichs), containing at times some fatty globules, some lymph corpuscles (Severi) or embryonal nuclei which form new connective tissue.

"Should the course of the disease be rapid, and death take place before the lesions of the secondary period become very marked, the liver would appear normal or almost so, chiefly when observed with the naked eye. (These are the conditions in which Dr. Quinquand has found an accumulation of extractive matter in the substance of the liver, well calculated to prove the existence of a real lesion.)

"*Third Period.*—Macroscopical and microscopical lesions of acute yellow atrophy at its maximum. During this period, as described in a former quotation, we may find on examining fresh slices with a microscope all the intermediary stages between the normal liver cells and the atrophied cell in process of disappearance, crystals of leucine and tyrosine are also met with. These products of disassimilation may exist during the preceding period. Appearance of new elements in the midst of connective tissue, the nature of which is not yet ascertained, which probably serve for the regeneration of liver cells.

"*Fourth Period.*—A tendency to reparation. Reparation of past disorders. Facts are wanting to answer with precision whether it is complete or limited."

Although this classification be artificial to some extent, although we know nothing of the period of reparation, and we do not know even if the lesions of the third period can be repaired, it gives with a good deal of truthfulness the sum of the lesions found and their habitual succession.

But the share referred to interstitial hepatitis is certainly too narrow, not because this increase of cellular tissue between the lobules has been very often observed, but because it shows, when well marked, the passage from acute to chronic atrophy. This leads us to think that if interstitial hepatitis is not more common, it is because it is not allowed the time to develop, the sustenance of life being incompatible with a total, or almost total, destruction of the liver cells; death supervenes before the prolific interstitial inflammations have time to be produced; neither is this a mere hypothesis, as we shall have soon an opportunity to prove that, in various poisonings, according to the dose of poison absorbed, we have acute yellow atrophy without interstitial hepatitis at times, at other times interstitial hepatitis together with atrophy, and finally we may sometimes have a chronic cirrhosis.

Winiwarter has found in a very acute case a considerable augmentation of the interstitial cellular tissue. The lymph corpuscles were exuded in large number into the acini and in their vicinity in the interlobular spaces were found fibrillæ and fusiform bodies. The liver cells were small, atrophied and cylindrical. Fick met a similar case. Lewitski and Brodowski have met with an infiltration of the cellular tissue of the liver with rounded cellular elements.

Finally, in some cases, there may be a proliferation of the smallest elements of the liver and of the intralobular capillaries. The biliary canaliculi are increased in size chiefly in the points of real atrophy. (Thierfelder.)

The inflammation of biliary ducts has been observed by a number of conscientious observers. Some have noticed an inflammation of the mucous membrane lining the ductus choledochus (Paulicki, Reiss), others found in the interior of the canal real plugs of mucus (Bamberger, Mann, Rosenstein, Davidson, etc.). But the biliary ducts sometimes contain no bile and are often filled with mucus, and a deeper cause remained to be found, to explain why the bile does not circulate. Buhl accepts the obliteration of the canaliculi as the cause of the jaundice. Bollinger and Perls agree with him. This obliteration might be owing to the compression due to the interstitial exudation when it exists (Frerichs), or to the accumulation of embryonal elements taking place at times in the spaces between the different structures. There is obliteration of the canaliculi from the cellular detritus furnished by the swollen walls, according to Bamberger. Thierfelder thinks that all these causes may act together.

The received notions regarding the etiology of common jaundice lead us to think that it is in the same manner a true resorption which may take place, very deep, as we can see in hypertrophic cirrhosis; the inflammation and the obstacle to excretion being seated near the lobules and the cells. This is important in another consideration still; it is sure that the degeneration of the liver cells, whence malignant jaundice results, has a certain activity which differentiates it from simple fatty infiltration, and this activity of the morbid process agrees well with the inflammation of the canaliculi.

Dr. Lancereaux lately published through his pupil, G. Dupont, an important thesis on acute diffuse interstitial hepatitis. Right or wrong, he attributes a great deal of importance in his work to the action of alcohol. However it may be, the observations recorded are most curious. In a clinical aspect, they recall all the features of malignant jaundice. Anatomically, a marked atrophy of the liver, which preserves its sharp edges, its yellow ochre hue, its elasticity, and a surface almost always glossy. "The microscope reveals in the whole extent of the viscus a diffuse infiltration of the parenchyma, beginning wherever the connective tissue exists in the liver, passing into the interior of the lobules between the liver cells, compressing and destroying the latter." These lesions recall very well those of syphilitic interstitial hepatitis, and have been carefully described by Dr. Rémy, who made an histological examination of them. Let us also remember that diffuse syphilitic interstitial hepatitis with an acute course is recognized as a cause of malignant jaundice.

In what does common cirrhosis, Laennec's cirrhosis, differ from hypertrophic cirrhosis? and why does it determine the appearance of the phenomena of malignant jaundice only in an advanced period, acting mechanically, as it were, after it has destroyed the liver cells already atrophied? This is because the feeble irritation long renewed has been acting at times on the capillaries of the vena portæ, at other times on the mucous lining of the biliary canaliculi. The liver cells were not affected at first because the irritation was too little, the blood holding the toxic matter in too small a quantity, or the poison being too feeble, could be noxious through repetition only. If on the contrary the dose of poison had been very large, or its irritating properties very active, the liver cells, attacked in all the extent of the liver, and more or less quickly destroyed, would cease their function and the symptoms of acute yellow atrophy would be manifested. This hypothesis can be directly demonstrated.

It is more and more generally admitted that phosphorus poisoning gives rise to malignant icterus. Considering the symptoms, the similarity is absolute, so also their pathological anatomy. Considered clinically, there is nothing in phosphorus poisoning which can distinguish it from malignant jaundice, the yellow tint of the teguments and of the urine. the emeses, often the hæmatemeses, diffuse hæmorrhages, nervous phenomena, are all present. Microscopical examination has not generally revealed atrophy of the liver; but this is not a characteristic, for, on the one hand acute hepatic atrophy is sometimes present in phosphorus poisoning, on the other, Liebermeister and Frerichs have found the liver normal in size in some well marked cases of malignant jaundice.

Those authors who do not admit the identity of malignant jaundice and phosphorus poisoning, rely chiefly on the fact that in malignant jaundice, there is a fatty granular degeneration of the cells, and in phosphorus poisoning a mere infiltration with fat. It seems to us that the existence, duly established, of irritative lesions, such as proliferative interstitial hepatitis, suffices to show in phosphorus poisoning something beside a simple infiltration, a passive deposit of fatty globules. It is very likely also that the jaundice of phosphorus poisoning can be referred to an inflammation of the canaliculi, as hinted by Ebstein and O. Wyss, though this remains yet to be demonstrated.

Some have looked for a chemical difference between fatty infiltration and degeneration. Perls pretends that whenever there is in a tissue a diminution of water with a corresponding increase of non volatile elements loaded with fat, there is infiltration.

There is degeneration on the other hand when, the quantity of water remaining normal, the fat replaces some other non-volatile elements. According to that, there would be degeneration instead of infiltration in phosphorus poisoning. Bauer and Voit tried to prove in another way that the presence of fat in the liver cells results from a destructive process in these. In order to prove that, they poisoned fasting dogs with phosphorus. The fat found in the liver cells must have proceeded from their own substance.

We acknowledge a preference for clinical demonstration, and we refer to the observation of Fraenkel for a proof that the lesions are a process of degeneration.

The macroscopical atrophy of the liver which evidently pertains to degeneration is clearly met in phosphorus poisoning (Thierfelder), and Fraenkel published in 1878 a case of acute poisoning observed at the clinique in Leyden. There was in the case a remarkable diminution in the size of the liver which had been observed before the occurence of death.

In this manner by modifying the dose of phosphorus, acute atrophy of the liver, and acute diffuse interstitial hepatitis can be produced at will, and if the doses are small and repeated, common cirrhosis will occur. Weil, giving heavy doses of phosphorus to dogs in order to kill them in twenty-four hours, found no lesions of the liver. With smaller doses he obtained a fatty infiltration at the anastomoses of the capillaries ; by a slower poisoning a very clear proliferation of the interstitial connective tissue with degeration of the liver cells.

Finally Wagner, who experimented with very small doses to act on the nutrition of the bones, met with common cirrhosis and even proved the secondary effect produced on the spleen and the blood vessels of the intestine, by the obstruction of the portal circulation in cirrhosis.

What results from the absorption of phosphorus through the digestive canal may be produced by a spontaneous poisoning of the blood from various causes.

Says Dr. Vulpian, the changes in the blood are the clearest and most constant signs of malignant jaundice. They consist in a disintegration. This carries us back to the hypothesis formerly sustained by Buhl and Trousseau, and we must admit that in the hæmorrhagic icterus of Monneret, and the malignant icterus of Ozanain, there is at first a general poisoning of the organism by an infectious principle of an unknown nature, and later a destruction of the liver with suspension of its activity. The liver cells, and more rarely the parenchyma, are affected by the zymotic poison carried in the blood, malignant jaundice soon develops, more or less rapidly, and more or less intense in its evolution according to the conditions of infection and resistance.

The true nature of that poison is unknown, resembling in this particular the contagium of typhoid fever. Klebs and Eppinger, who met with bacteria in the blood, charge them with causing the disease. This conclusion is premature since the presence of bacteria is not pecular to malignant jaundice. It is remarkable that acute yellow atrophy and the phenomena of malignant jaundice may be produced in the course of typhus fever, of typhoid fever, and other acute affections. In all febrile diseases, the liver may undergo a true, acute fatty degeneration. Between this degeneration, more or less marked, and acute yellow atrophy there is no well-defined line of demarcation. Indeed, we find some cases recorded in which liver lesion, following a pyrexia foreign to essential hæmorrhagic jaundice, did not differ from the lesion of acute yellow atrophy. This hepatic degeneration has chiefly been met with in typhoid fever. (Frerichs, Oppolzer, Hoffmann, Eppinger.) Diett, Chedevergue, Griessinger and Murchison have recorded cases in which the jaundice supervened in the course of typhoid fever or during a grave relapse.

Dr. Mossé quotes an observation of Dr. Sabourin : a typhoid patient dying with a true acute yellow atrophy. In reality we must admit that, despite the exception, acute yellow atrophy may arise in the course of typhoid fever, and according to Dr. Mossé, during pneumonia. This is not to be wondered at if we consider that malignant jaundice is simply the result of a destruction going on in the liver, whatever the original cause may be.

The existence of a poison whose mode of action is reproduced by phosphorus, cannot be denied since malignant jaundice may occur in epidemics.

Such epidemics have occurred in a sufficient number. They seem to rage in two circumstances chiefly. Sometimes there is an epidemic of mild jaundice occurring as a mild disease, taking with a few a malignant character. The danger seems to result from a want of resistance in the individuals attacked, rather than from a peculiarly malignant character of the infection.

The epidemic lately recorded by Frohlich seems to belong to that order. At other times, on the other hand, it is malignant in itself. All the individulals attacked are profoundly affected from the start; a cure becomes the exception. In this class belongs the epidemic of Lille, narrated by Coyne and Arnould. Two patients only escaped. All the rest died with the symptoms of malignant jaundice.

The want of resistance is particularly marked in pregnant women. During epidemics, even relatively mild, pregnant women and those lately confined, almost always furnished the disease with an important material, and deaths were common. It has even at times attacked pregnant women exclusively. Should we look to the fatty infiltration of the liver, normal under those circumstances, as a predisposing cause to the development of the lesions of malignant icterus? That is possible, and there is probably no line of demarcation between this physiological fatty infiltration and a morbid atrophy.

Then it is evident that essential malignant jaundice is owing to a general poisoning of the organism. The blood in this affection is in a disintegrated state as in all infectious diseases. It is black, fluid, dissolved, in a word, and presents very little propensity to coagulate. This peculiar fluid state doubtless facilitates its exudation through the vessels. "The red corpuscles are diminished in the number; part of them have been dissolved, and many of those that remain have lost their shape and become spheroidal; the serum is reddish, and the blood resembles wine dregs in color." Vulpian, Riess, Frerichs, Quinquand, have either found leucine and tyrosine, or extractive matter nearly allied, in the blood. If we remember that leucine and tyrosine have often been found in the liver, or in other organs, the brain for instance, and that they have been found in the urine about as often as they were looked after, we must give them some significance. But it is more probable that they are the result rather than the cause of the disintegration of the liver.

The appearance of malignant icterus as an epidemic in temperate regions, leads us to consider a question long debated, and very differently solved by authors. Are icterus and yellow fever identical? is malignant jaundice, as Monneret said, nothing but the yellow fever of our climates? We dare not discuss the question for want of facts to solve it. Let us remember, however, that Griesinger, after long argument to prove that a wide difference exists in the two diseases, concludes by admitting that malignant icterus occurring in intertropical countries could hardly be differentiated from yellow fever. An important communication was made in 1877 by Dr. Lebredo, of Cuba, to the *Société de Biologie.* This writer having made an histological examination of the liver in two cases of yellow fever, found cirrhosis disseminated in spots, like common cirrhosis of a bilious origin; round particles analogous to yellow embryonal cells in the spaces of Kiernan, and, lastly, a marked atrophy of the liver cells with an extensive fatty degeneration. According to this view, there would be no difference between the lesions in the black vomit and in malignant jaundice.

Should an identity of yellow fever and malignant jaundice be ever demonstrated, it would be satisfactory to see how, by an ascending and continuous progression, the signification of the complex malignant jaundice, in a continuous and progressive ascension, becomes more and more prominent, how it tends to take the first rank from the symptomatic malignant icterus of a chronic and morbid process in the liver to yellow fever; how the same complexus of manifestations, with a variable intensity, is produced now after a poisoning slowly and indirectly destructive, alcoholism for instance, at other times during a rapid poisoning capable of generating epidemics and of transmission by contagion.

However attractive this view may appear, it is not yet demonstrated, at least in what relates to yellow fever.

It remains, before closing this review, to bring forward an interesting point in the history of malignant jaundice, the possibility of its resulting in a cure. Until later times, death was considered a necessary termination, and, some years ago, Grellety-Boswell, who saw many cases terminating in a cure, durst not, for that very reason, admit the existence of malignant jaundice in these cases. He proposed to call it pseudo-malignant icterus.

The case of Dr. Bouchard, above quoted, is one of the clearest that could be met with. Nothing was wanting to the description of malignant jaundice in that case, though the patient recovered. The quantity of urine had been regularly measured, the urea carefully weighed. In a first period there was an augmentation in the quantity of both. In a second period, a marked diminution; and lastly, as a crisis, the flow of urine became profuse. Similar facts were recorded by Mossé, Arnould, and Coyne, and the rise in the quantity of urine and urea has always been the first step to convalescence. This indication, we see, is a precious one to remember.

If, before closing, a definition of malignant jaundice has to be formulated, a short description stating its nature and its character, we may say: Malignant jaundice is a complexus of symptoms consisting essentially of cerebral phenomena, coma, stupor, delirium, convulsions, various hæmorrhages, and generally, though not necessarily, of jaundice. This complexus has its cause in the irritative destruction of the cells of the liver, whatever the origin or the rapidity of that destruction may be, provided it involves the liver to a certain extent. Finally, the presence of malignant jaundice does not necessarily indicate a fatal prognosis.

---

INHALATION OF OXYGEN IN VOMITING OF PREGNANCY.—M. Pinard publishes in the *Ann. de Gynecologie* an interesting case of obstinate vomiting cured by inhalations of oxygen. It was the case of a young woman who had reached the fourth month of pregnancy and vomited constantly. She had suffered considerable emaciation without having reached the febrile period. Treatment had consisted especially in opium internally, subcutaneous injections of morphia and the ether spray. For three days in succession she was made to inhale oxygen, ten liters the first day, twelve liters the second day, and fifteen liters the third. From this day the vomiting ceased and pregnancy went on to term.—*Journal de Médicine*, p. 372, Aug., 1880.

